# Supplementary material for: The Relation between Plasma Nesfatin-1 Levels and Aggressive Behavior in Pit Bull Dogs
Source: Animals (Basel). 2024 Feb 16;14(4):632. doi: 10.3390/ani14040632 (PMC10886264; doi:10.3390/ani14040632)
Supplement: Supplementary file 1 [file animals-14-00632-s001.zip › Aggression_Test.pdf]

Aggression Test

Ear/cage number:

Race:

Gender:

Age:

Arrival date:

Neutered date:

Known acute or chronic illness:

Known incidents of violence (biting, dog fighting, harming employees, etc.):
Body mass index (numbering 1-5) (muscle condition and weight height should be considered):

	Questions	Never	Rarely	Often	Always
**1**	Aggressive when its food is touched while it is eating	①	②	③	④
**2**	Aggressive when it is passed while eating	①	②	③	④
**3**	Aggressive when food is added to its bowl while eating	①	②	③	④
**4**	Aggressive when its food is taken	①	②	③	④
**5**	Aggressive when awakened by physical contact	①	②	③	④
**6**	Aggressive when physically disturbed at rest	①	②	③	④
**7**	Aggressive when attempting to get it out of the cage	①	②	③	④
**8**	Aggressive with more than 5 seconds of eye contact	①	②	③	④
**9**	Aggressive at grooming, nail clipping, ear cleaning, or other painless procedures	①	②	③	④
**10**	Aggressive when grabbed by the leash and pulled back	①	②	③	④
**11**	Aggressive when removing or putting on the collar	①	②	③	④
**12**	Aggressive to loud sounds	①	②	③	④
**13**	Aggressive when threatened from afar by showing a newspaper, stick, or hand	①	②	③	④
**14**	Aggressive when walking next to it in the cage	①	②	③	④
**15**	Aggressive when asked to respond to commands	①	②	③	④
**16**	It wants to go to a determined place when it leaves the cage	①	②	③	④
**17**	Aggressive when unfollowing its path	①	②	③	④
**18**	Overprotective toward those it loves.	①	②	③	④
**19**	Aggressive when a stranger touches loved ones	①	②	③	④
**20**	Aggressive when accidentally pressed on its tail or one of its limbs	①	②	③	④
**21**	Aggressive when a stranger approaches	①	②	③	④
**22**	Responds to restlessness, loss of appetite, tremors, and excessive salivation when the keeper leaves it	①	②	③	④
